# A midas plugin to enable construction of reproducible web-based image processing pipelines

**DOI:** 10.3389/fninf.2013.00046

**Published:** 2013-12-30

**Authors:** Michael Grauer, Patrick Reynolds, Marion Hoogstoel, Francois Budin, Martin A. Styner, Ipek Oguz

**Affiliations:** ^1^Kitware, Inc.Carrboro NC, USA; ^2^Neuro Image Research and Analysis Laboratories, Department of Psychiatry, University of North CarolinaChapel Hill, NC, USA; ^3^Department of Electrical-Computer Engineering, University of IowaIowa City, IA, USA

**Keywords:** brain image processing, automated pipelines, rodent imaging, MRI, workflow processing

## Abstract

Image processing is an important quantitative technique for neuroscience researchers, but difficult for those who lack experience in the field. In this paper we present a web-based platform that allows an expert to create a brain image processing pipeline, enabling execution of that pipeline even by those biomedical researchers with limited image processing knowledge. These tools are implemented as a plugin for Midas, an open-source toolkit for creating web based scientific data storage and processing platforms. Using this plugin, an image processing expert can construct a pipeline, create a web-based User Interface, manage jobs, and visualize intermediate results. Pipelines are executed on a grid computing platform using BatchMake and HTCondor. This represents a new capability for biomedical researchers and offers an innovative platform for scientific collaboration. Current tools work well, but can be inaccessible for those lacking image processing expertise. Using this plugin, researchers in collaboration with image processing experts can create workflows with reasonable default settings and streamlined user interfaces, and data can be processed easily from a lab environment without the need for a powerful desktop computer. This platform allows simplified troubleshooting, centralized maintenance, and easy data sharing with collaborators. These capabilities enable reproducible science by sharing datasets and processing pipelines between collaborators. In this paper, we present a description of this innovative Midas plugin, along with results obtained from building and executing several ITK based image processing workflows for diffusion weighted MRI (DW MRI) of rodent brain images, as well as recommendations for building automated image processing pipelines. Although the particular image processing pipelines developed were focused on rodent brain MRI, the presented plugin can be used to support any executable or script-based pipeline.

## Introduction

Magnetic Resonance Imaging (MRI) is a valuable method for analyzing neuro-anatomical structures and connectivity. Structural Magnetic Resonance Imaging (sMRI) and Diffusion Tensor MRI (DTI) are two common imaging types used in such studies. MRI is highly useful because the method is non-invasive, has no known biological effects on subject tissues, and can acquire data in similar ways for human and animal subjects. Neuroscientists often use the translational capabilities of MR imaging to study rodent models of human neurological and psychiatric conditions.

A common image analysis study design in this context would consist of acquiring brain MRI of the experimental and control groups, then performing region-based analyses of the MR images to compare the populations. An example of these region-based analyses would be finding the average and standard deviation of the volume and intensity of specific regions in the brain after they have been segmented. While it is possible to perform these steps manually, this is labor intensive and often results in subjective variability that precludes reproducible analyses. These problems led to the recognition of the need for a software system that enables the construction of automated, reproducible neuro-image processing pipelines for rodent brain MR data. In order to develop and test the pipelines quickly, the system needed to provide server-side processing running on a computational grid infrastructure, co-located with the centralized data archive Midas where our study results were stored. These pipelines needed to be modifiable throughout their development, to present common graphical user interfaces across different platforms, and be straightforward to develop for image processing experts but also easy to use for clinical collaborators lacking this expertise. These pipelines would be common tools presented to both the image processing experts and the clinical collaborators, and would run in the exact same computational environment and using the same datasets independent of the user triggering a run. The image processing experts needed to be able to build and tune the pipelines, providing both appropriate parameters for the pipelines as well as interpretation of results for their collaborators.

There are existing software tools for carrying out image processing tasks or for building these into pipelines, such as Slicer^1^ (Pieper et al., 2004), FSL^2^ (Smith et al., 2004), SPM^3^, and Nipype^4^ (Gorgolewski et al., 2011). However, these are complex to use, especially for someone lacking image processing knowledge. They do not always provide the correct set of default parameter values; for instance, Slicer has a registration module that has good default parameter values for human brain MRI, but these are inappropriate for use with rodent brain images. Furthermore, these tools typically rely on local data, which introduces problems of locating and downloading the data, identifying its provenance (e.g., raw data from a scanner in a particular study vs. the result of various processing steps previously carried out with a particular set of parameters), and sharing that data with collaborators. Using these tools also makes it difficult to reproduce a processing experiment, as there often is no good mechanism for sharing parameter values outside of a local instance of the software. The software setup, because these are local tools, is dependent on the individual environment of the machine, which can introduce subtle changes between setups that are difficult to identify. Results produced by a researcher are local to that researcher and cannot easily be shared or compared against subsequent runs by other researchers. At the time our project commenced, we could find no known systems available to the neuroscience community that adequately solved all of the above issues, hence the need to create just such a system using Midas. Developing pipelines for our system can be technically involved like these existing systems, but with the centralized storage and sharing of data, and separation of development from web interface presentation, programmers can develop pipelines that can then be used by non-programmer collaborators, dramatically reducing the use-of-complexity for this latter group. We recognize that inevitably, there will be other groups who decide to build their own pipeline systems rather than using existing systems. For these researchers we provide recommendations for building automated image processing pipelines below.

Midas^5^ is an open-source toolkit for creating web based scientific data storage and processing platforms. It was created to solve the problems of archiving, managing, and distributing image processing datasets. These datasets are large in file size, need to have variable access controls, and are essential for sharing throughout the image processing research community as a base for research and to ensure scientific reproducibility. Midas can be extended through plugin modules to allow customized data analysis, visualization, and server-side processing.

Midas is easy to use, even for non-programmers such as biological and clinical researchers, and it allows them to share data with their collaborators. The flexible group and permission system allows control over whom the data is shared with. By providing a centralized data resource, there is an authoritative version of the data that can be tracked over time for changes. The plugin system allows for significantly enhanced functionality over the stock Midas system, such as visualizing imaging results in the browser or performing server-side processing. In this paper, we present a specific application of the plugin system, describing how it allows for creating image processing pipelines by experts familiar with these techniques and making these available to collaborators who may lack this expertise.

The Rodent Imaging plugin for Midas that we describe below allows an image processing expert to construct a pipeline of executables, create a web-based user interface, manage jobs, visualize intermediate results and create compositions of multiple pipelines. Since input data and output data are centrally stored, the user experience for sharing with collaborators is tremendously improved and simplified. Collaborators who are not image processing programmers are also given an opportunity to run complex image processing tools without having to understand command line execution or complex file operations. Furthermore, the users can follow the results of the image processing pipelines as they are brought back into Midas, preview the results in the web, and can share these results with other researchers. The computational environment and set of parameters are also shared and available to all collaborators. As the three inputs to image processing—parameters, input data and computational environment—are shared between researchers, this system yields greater reproducibility of results, which is a core principle of scientific research.

It should be noted that the design of the actual image processing pipeline itself, such as the choice of particular registration and segmentation methods to use, is beyond the scope of this current manuscript. This image processing pipeline created with this plugin and the outputs of running the pipeline are described elsewhere (Budin et al., 2013) and the individual components were described and validated in earlier work (Lee et al., 2009, 2011; Oguzet al., 2011; Rumpleet al., 2013). In this paper, we focus on describing a novel plugin to the Midas platform that enables the image processing experts to port such a pipeline into Midas, creating a streamlined web-based user interface.

The rest of this paper is organized as follows. In Section Materials and Methods, we describe the Midas system and the Midas plugin for developing image processing algorithms, followed by an example walk-through of preparing an ITK-based application for grid execution and generating a pipeline user interface (UI) for the application. In Section Results, we present the pipelines we developed using this plugin and the results of running the pipelines to illustrate the performance of our platform. Finally, in Section Discussion, we discuss our experience with developing this pipeline along with possible improvements, and present guidelines for developers creating automated image processing pipelines.

## Materials and methods

### Midas

**Figure 1:**
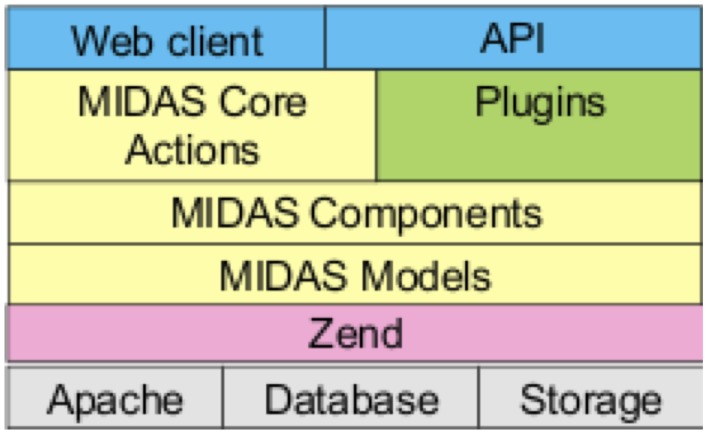
**Midas architecture**.

Midas is an open source software platform that provides scientific data management services, especially for large image data (Jomier et al., 2009, 2010, 2011). Written in PHP^6^, its architecture, shown in Figure 1, is based on the Zend^7^ application framework, typically running in an Apache^8^ web server, using either a Mysql^9^ or PostGreSql^10^ database backend, with search support from the Apache Lucene^11^ project. The Midas Platform provides a core set of functionality for organizing and managing files and folders in a hierarchical structure, and provides a framework for data access through various methods (including web, filesystem and DICOM server). It also provides for user and group creation, and a permissions structure similar to the unix filesystem permissions that allows for read/write/ownership permissions on individual files and folders. Any given resource in Midas, whether a folder or a file, can be viewable by anonymous users (those that have not logged in), or restricted to only be viewable by users given certain permissions.

Upon creating a user account, a user of Midas is given a public and private folder, with appropriate permissions corresponding to each. The user can then add additional folders and files under those folders, and change permissions of any folders or files.

In addition to user spaces, there are spaces called communities, which are shared spaces between users. The notion of a community is of a top-level point to add folders and files that are organized around a single group of users or purpose. Communities can have folders that are publicly available to anonymous users, or can be restricted to logged in users, or to only those users that are members of the community, moderators of the community, or administrators of the community.

All of the operations relating to the resources in Midas are available through a RESTful^12^ API, which provides the ability to create, read, update and delete resources via specific URLs. There is a Python client library Pydas^13^ that wraps much of this API functionality; this library allows for programmatic interaction with a Midas server.

Midas is architected to allow for expansion through plugins, which can be turned on or off to suit a particular setup. The plugins have access to all the core resources and functionality as well as the core views. Plugins can add features to the core views and add their own views, and add their functionality to the core RESTful API set.

### Midas image processing pipeline plugin

In order to construct automated pipelines for image analysis, we have built a Midas plugin^14^ that allows for the construction of pipelines based upon configuration. The inputs for a pipeline are a set of command line executables (statically linked), a BatchMake script to coordinate inputs and outputs for the executables, and a configuration file to describe the UI displayed within Midas and parameter routing.

To develop a particular pipeline, a BatchMake^15^ script is developed that includes one or more command line executables and their parameters. BatchMake is a scripting language that allows for local execution of a pipeline as well as execution under the HTCondor^16^ grid computing environment. Local execution allows for debugging and rapid iteration, whereas execution under the HTCondor environment allows for arbitrarily large scalability (within the limits of parallelism of the pipeline).

Here, we describe the procedure for building an ITK-based executable and wrapping it for BatchMake and HTCondor grid execution. For the sake of clarity, we illustrate these concepts using a simple executable that applies a Gaussian smoothing filter to the input image. The rodent brain imaging pipeline that we have developed is considerably more complex, as can be expected; however, it is based on the same principles we discuss in detail and any noteworthy differences are pointed out.

#### An example of preparing an ITK-based application for grid execution

**Figure 2:**
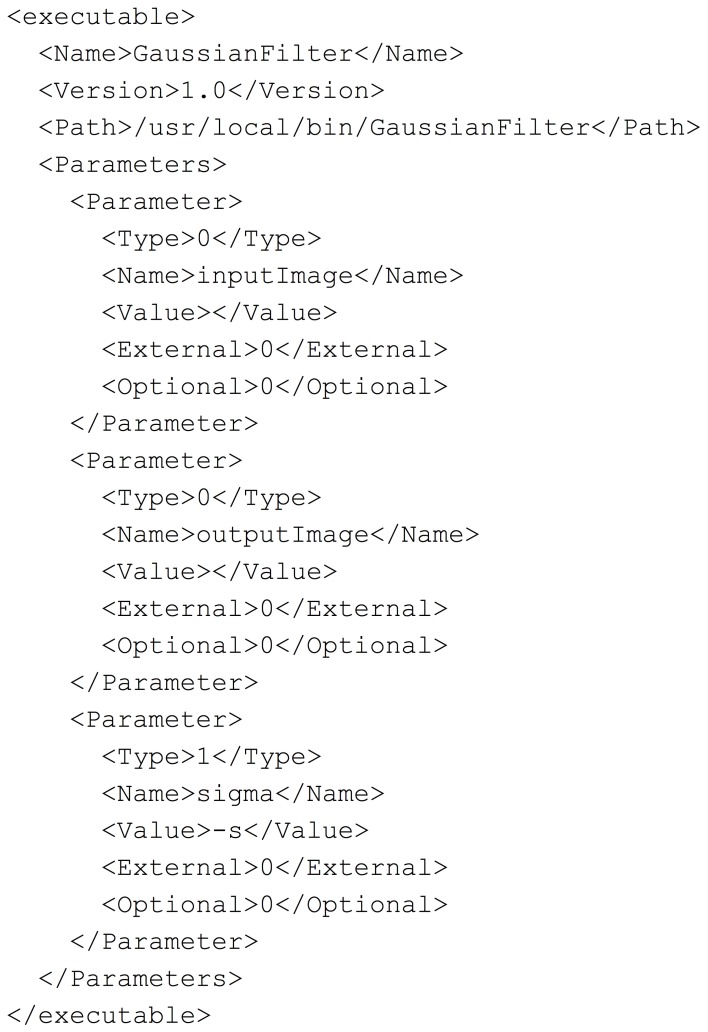
**GaussianFilter.bmm**.

To demonstrate parts of the pipeline development process, we have created a simple ITK based application to perform a Gaussian smoothing on an image. In order for BatchMake to create HTCondor grid jobs for this executable, we need to define the command line parameters of the executable in a.bmm file. In the case of a Slicer-compatible executable, the data for the.bmm can be readily derived from the XML description used by the GenerateCLP library in the Slicer Execution Model. The.bmm file shown in Figure 2 defines the inputs to the GaussianFilter executable as a string that is the name of an input image file, a string that is the name of the output image file, and a value for σ, the size of the Gaussian kernel. The Name element allows BatchMake scripts to refer to this application and the Path element indicates the full path to the executable on the executing nodes of the HTCondor grid, which may be different from the job submission or grid manager node. The Value element indicates what value to print when the parameter is invoked, beyond the parameter value itself; e.g., in the case of the σ parameter, a “-s” will precede the actual chosen value for σ, thus passing in the correct command line flag to the executable.

**Figure 3:**
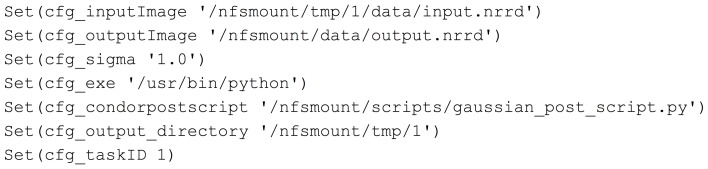
**Gaussian.config.bms**.

Midas and the image processing pipeline plugin define and create configuration files that will be included by a BatchMake script (bms file). This is how user selections and configuration settings specified in the web application will be delivered to the command line executables. In this example, we have created the contents of a configuration file gaussian.config.bms in Figure 3 that includes parameters passed from Midas. Here the path of the input and output images are passed, along with the value for the σ parameter, the executable to run the HTCondor post-script, and the output directory for the command line executable. When a processing request is made from Midas, a temporary work directory is created (cfg_output_directory) on the submitting machine. Any input data (cfg_inputImage) is linked from Midas into the work directory, and the executable is instructed to create the output image in that same work directory. Note that the input and output files are created in locations that are available to both the submitting machine and the executing machine in the HTCondor grid. The cfg_exe will be run as the HTCondor Post-script, with the argument of the Python script file itself, gaussian_post_script.py. The HTCondor Post- and Pre-scripts are always executed on the submitting machine. Further arguments to the Python script file itself could be easily added to the pipeline BatchMake script.

**Figure 4:**
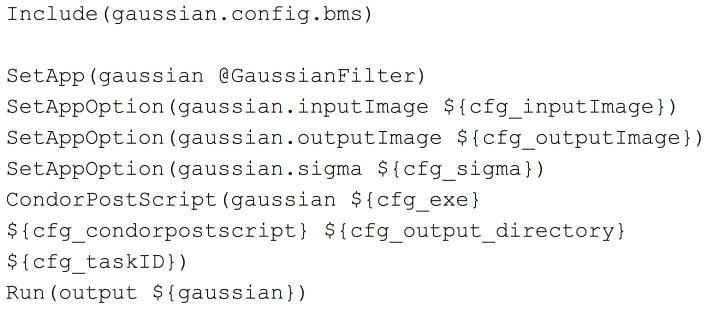
**GaussianFilter.bms**.

The pipeline BatchMake script GaussianFilter.bms, shown in Figure 4, then includes the configuration BatchMake script to obtain these parameter values specific to each individual job. The application gaussian is set to GaussianFilter, which was defined in GaussianFilter.bmm to have parameters of inputImage, outputImage and sigma. The CondorPostScript command will create a POST script in the HTCondor DAG with the given parameters.

**Figure 5:**
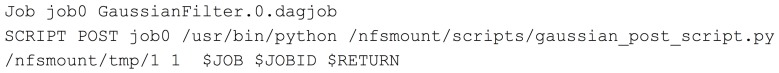
**GaussianFilter.bms generated DAG file contents**.

The BatchMake executable runs with parameters GaussianFilter.bmm and GaussianFilter.bms to create a HTCondor DAG (Directed Acyclic Graph) file and individual job files, which are created in the work directory on the submitting machine. DAGs are commonly used to describe workflow processes, with nodes of the graph representing steps in the workflow, and directed edges between nodes showing time order dependency between the steps. Because the graph is acyclic, there is a known starting point and the workflow cannot loop, meaning it must finish after each individual step is finished. The contents of the DAG file for our example are shown in Figure 5. Only a single job, job0 is created in this very simple scenario; however, multiple jobs could be created by a more complex BatchMake script with the sequencing dependencies between the jobs expressed in the DAG file. For instance, if two executables need to be called in sequence, there would be a dependency between them with the job corresponding to the second executable being the child of the job corresponding to the first executable. Executable jobs that are created in a loop by different iterations of the loop can occur in parallel and therefore have no ordering dependency between them. This DAG file example also contains the HTCondor POST script command with its parameters.

**Figure 6:**
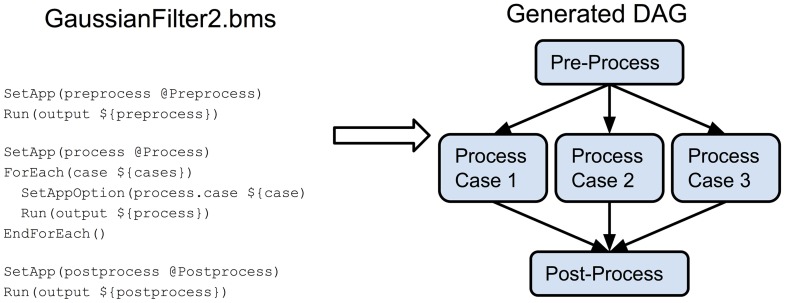
**GaussianFilter2.bms and generated DAG**.

An example of constructing a more complex pipeline is presented in the GaussianFilter2.bms BatchMake script in Figure 6. In this case, we have a pre-processing step for the entire dataset, followed by a processing step that would run on each input case, with a final post-processing step for the entire dataset. Since the three steps occur in sequence, there is an ordering dependency between them. For the processing step itself, since it occurs within a loop, the individual processing jobs (the three separate cases) can be parallelized. A graphical representation of the resulting DAG is also shown in Figure 6.

**Figure 7:**
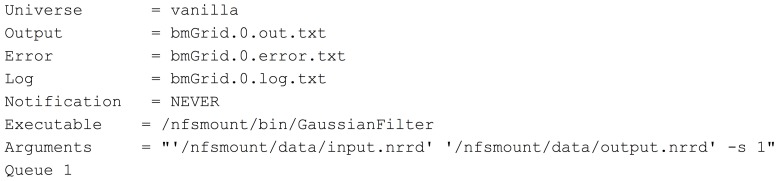
**GaussianFilter.0.dagjob**.

Returning to the simpler GaussianFilter example, the file GaussianFilter.0.dagjob shown in Figure 7, is automatically created in the work directory on the submitting machine, along with any other individual job files. This file specifies the actual execution that will be run by HTCondor on whichever executing machine in the HTCondor grid will run this particular job. The POST script will be run on the submitting machine after the individual job, and will take in the return value from the executable job.

**Figure 8:**
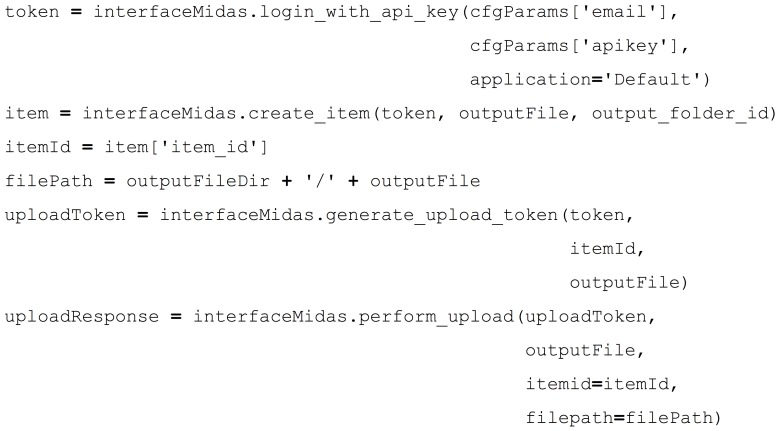
**Post-processing Python script**.

After execution of the GaussianFilter, the path to the output file will be passed to a Python script which will then upload the output file to the proper place in Midas. Using the HTCondor PRE and POST scripts, status and progress of the overall DAG and individual jobs in the DAG can be tracked from Midas and made available to the user. This way the user can view the status of the actual pipeline as it is processing on the HTCondor grid, from the web application. The standard output and standard error logs of the executable are also uploaded to Midas to enable executable debugging via the web interface. The Python scripts for our actual pipelines make extensive use of the Pydas library, a Midas Python client library that enables calling Midas' API endpoints. The Python code in Figure 8 shows an example of uploading an output file.

Once an image processing pipeline has been developed and its input data have been added to a Midas instance, a pipeline UI within Midas is created through a configuration file. The UI generated from this configuration file presents options to the end user of the pipeline to select among inputs determined by the configuration file, and then starts a processing job. At this time Midas will export all data necessary for the pipeline, and execute the BatchMake pipeline via HTCondor, which will then upload results to an output folder that was determined by the pipeline configuration file. An example of generating a pipeline UI follows.

#### An example of an automatically generated pipeline UI

Beyond the example of the GaussianFilter presented above, we will demonstrate our work in generating UI components for a pipeline with examples from the Registration component of the rodent brain image processing pipeline developed in our plugin. One of the innovative contributions of this work is bringing the concept of automated user interface components to the neuroimaging field.

**Figure 9:**
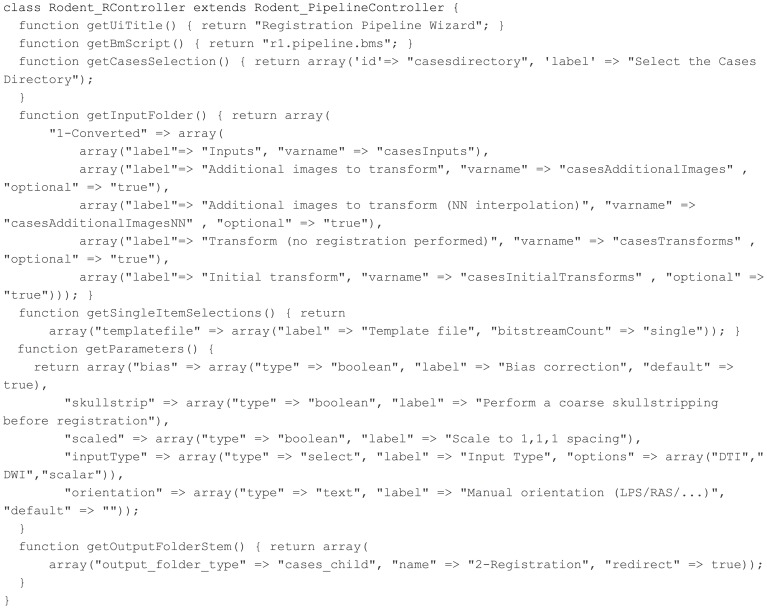
**Registration pipeline controller**.

Our goal is to allow for ease of development of different pipelines within Midas by developers without much Midas experience. For this reason, we created a Rodent_PipelineController PHP superclass that does most of the work of creating interface components for a pipeline and handing off parameters to the BatchMake and HTCondor components. To add a new pipeline, the developer must simply create a new controller in PHP extending this superclass; we present an example of this in Figure 9.

Then this specific pipeline controller class implements abstract methods from the superclass to define general parameters for the pipeline such as the title to display on the UI and the name of the BatchMake script to run. The BatchMake script implements the logic of creating jobs for the command line executables based on the passed in parameters, similar to the simple GaussianFilter example described above.

**Figure 10:**
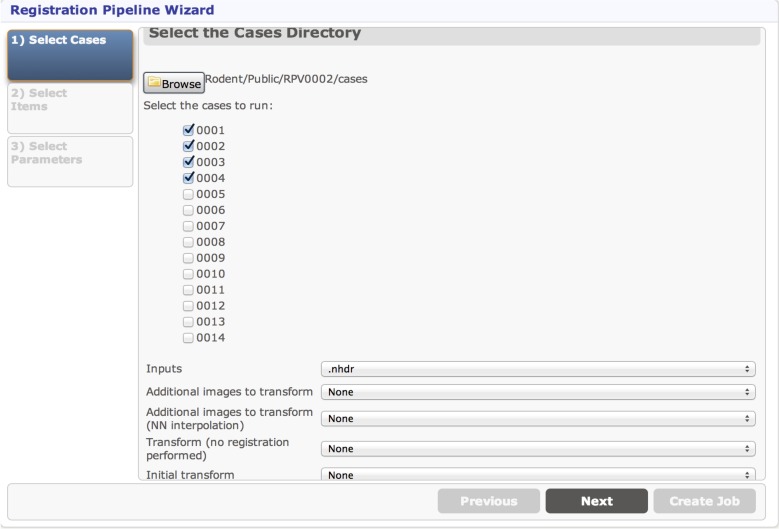
**Cases selection page**.

The first page in the UI for the Registration pipeline is the selection of input cases. The values set in the getCasesSelection method determine the default folder to choose cases from, but a different folder can be selected with the Browse button. The cases that are presented as checkboxes for the user to select from come from the Midas folder named 1-Converted, a subfolder of the cases folder. The Inputs parameter allows the user to select a filename pattern from a dropdown for the actual input to this pipeline, and the additional dropdowns are populated to allow the user to select additional files coming from each of the selected case directories, which gives the ability to send multiple input files to be processed together as part of the same case. The cases selection page created by these inputs is displayed in Figure 10.

**Figure 11:**
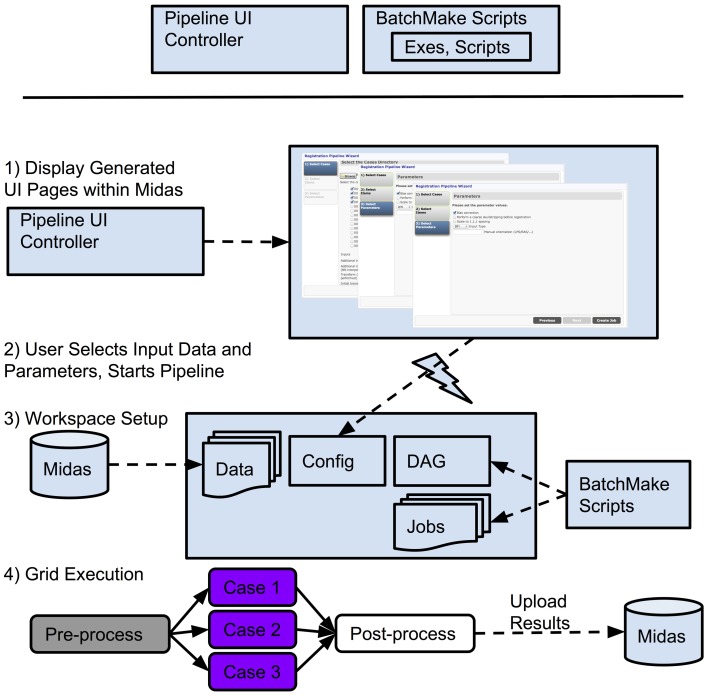
**Pipeline development and execution**.

The second page in the Registration pipeline UI is determined by the getSingleItemSelections method. This allows the user to select a single Midas item (file) to be used in processing all cases. The resulting UI page is similar to that displayed in Figure 10 and can be seen in step (1) of Figure 11. The final UI page of the Registration pipeline allows the user to select parameters, and will supply default values based on what is implemented in the getParameters method. The parameter selection page for the Registration pipeline is similar to that displayed in Figure 10 and can be seen in step (1) of Figure 11.

The getOutputFolderStem method will determine where the outputs of the Registration pipeline will be uploaded back into Midas when processing is complete. Here cases_child indicates that a new folder name 2-Registration that is a child folder of the case folder will be created for each case the user selected for processing, and this will be the location where outputs are uploaded when that case is processed.

#### Putting it all together

We will now describe an example of an overall pipeline execution based on Figure 11. Once the developers have created BatchMake scripts for the pipeline and the Pipeline UI controller, the pipeline is available for an end user. In step (1), the user views the pipeline UI presented through Midas, with the UI pages automatically created based upon the Pipeline UI controller. Based on the configuration file for the pipeline, the user will be presented in step (2) with some options for setting parameters and selecting input data that is stored in Midas, or can simply choose default values if they have been set for the pipeline, and can then request execution. In step (3), the Midas system will create a temporary workspace for this pipeline execution on the associated filesystem, and the data referenced by this execution of the pipeline is symlinked into this temporary directory. The parameter values for this run of the pipeline are exported into this work directory for inclusion by the BatchMake script. The BatchMake script is then compiled and converted into HTCondor job description files and an HTCondor DAG file. There will be one HTCondor job description file for each instantiation of a command line executable within the BatchMake script, and the DAG specifies the dependencies between the jobs. In step (4), the HTCondor DAG is submitted to the HTCondor grid, which allows for the execution of the pipeline to be scaled up to the limits of available hardware or budget. As each step of the pipeline finishes execution, a post-processing script can optionally be run, e.g., the Python script described above. For the pipelines we implemented, these post-processing steps are used to load intermediate files, result files, and metadata produced by the pipeline back into Midas. Midas has a plugin to visualize any image format that ITK can read, and as a result, end users can see the intermediate and final results of their pipelines displayed within Midas itself as the results come back.

Once a pipeline has been developed, an end user can run them even if that user has no image processing experience, and can run them in a completely reproducible manner. After creation of pipelines, pipeline composition becomes possible, so that a user can run multiple pipelines in sequence on a given dataset, changing parameters in order to visualize how this will affect the results. The user may also run pipelines using any of several outputs from a previous step, enabling them to see how changes in one stage of the pipeline affect a later stage before accepting a single output of a stage as the canonical input for the following stage or the final result, once that output has passed manual quality assurance tests.

## Results

The Midas Rodent plugin was developed to enable creation of image analysis pipelines, and was used specifically to construct a composite pipeline that perform region-based analysis of 3D rodent brain MRI images. This pipeline and the results of running it on our input datasets are described in detail in (Budin et al., 2013). This composite pipeline is composed of six smaller pipelines, and can process DWI, DTI and structural MRI data. The pipeline includes both pre-existing tools and tools developed for this pipeline. The pipeline comprises six major steps: (1) rigid registration, (2) skull-stripping, (3) population average creation, (4) population average segmentation, (5) segmentation propagation to individual subjects, and (6) region based statistics. The output of each step is used as the input to the subsequent step. The inputs to the overall pipeline are images acquired by MRI, and then stored in Midas. The inputs are converted from DICOM or raw formats to ITK readable formats such as Meta, Analyze, Nifti, and Nrrd, since most of the executables within the pipeline are built on top of ITK.

**Figure 12:**
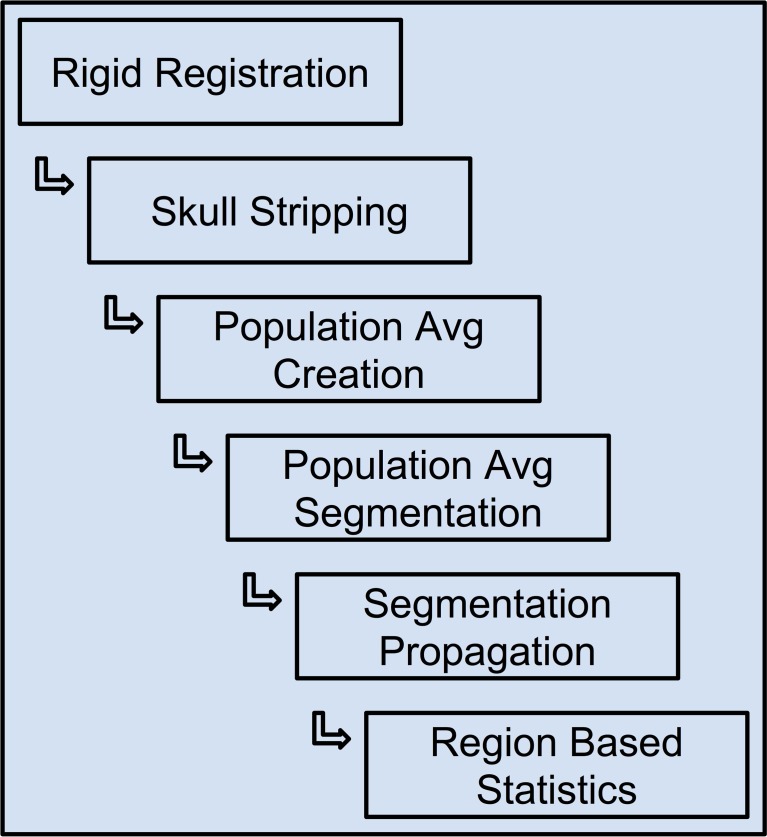
**The constructed pipelines used for rodent brain image processing**.

Each of the pipelines was built and debugged separately, and is designed to be run independently of the others. Once a given pipeline is debugged and validated, we can use them to process our subject cases. The output of a pipeline step, once deemed acceptable, becomes the input to the succeeding step. We may sometimes need to reprocess a given pipeline many times to achieve the correct parameter set, or to compare the outputs generated with different parameters or even alternative pipelines. This can be viewed as a data quality assurance process. After multiple runs of a given pipeline, the outputs of each of those runs—which would vary based on parameter selection—can be examined for quality and potential errors. Once the output of a certain run is approved, it can be used as the canonical input to the following step. Since the parameters of each pipeline run are tracked, it is straightforward to find the parameters that went into creating any particular output run of a pipeline, and hence that same pipeline run is reproducible. This illustrates how using our system seamlessly resolves the data provenance problem that often causes issues in large or even middle-scale studies. Figure 12 shows the flow of data from one pipeline to the next.

Due to running the pipelines on the grid system, we were able to execute the cases in parallel, which saved significant wall-clock running time. This also helped to develop and debug the pipelines faster, as we could see the results from a pipeline run much more quickly than if we had to run each step in series. However, there was a cost in development time, as the additional layers of preparing the executables for grid execution incurred overhead compared to simply running the executables locally. Debugging in the HTCondor environment is not as straightforward as debugging an executable on its own. There could be bugs in the executables, bmm file, the logic of the BatchMake script, the networked file system or HTCondor grid setup, and the multi-layered interaction between these components added complexity. Once a pipeline is developed, it enables much easier development of subsequent pipelines, as inputs could be quickly rerun to evaluate parameter choices and their effects on the later pipelines.

The centralized nature of this platform was helpful in development from the standpoint of data sharing, since multiple developers were working together on different pipelines and aspects of the overall system. As there was only the one Midas repository for data outputs, one developer could run a pipeline and ask another developer to look at the outputs without having to send the data. A developer could also easily use the output from another developer's run of a pipeline as an input to their pipeline.

## Discussion

This web-based system for running complex multi-step image processing pipelines enables researchers with little computational image processing expertise to access cutting-edge methods for segmentation, registration, and statistical analysis of rodent dMRI imagery, but can potentially be extended to processing of human imagery using similar methods. This system does not currently have a formal facility for manual intervention. If a manual step in a pipeline is desired, that could be achieved by breaking the pipeline into two sub-stages around the manual step. The output of the first sub-stage could be downloaded by the user, then used as the basis for a manual processing step (e.g., manual ROI, manual registration, etc.), and then the result of that manual step can be uploaded and used as the input to the second sub-stage. It would also be possible to add these “breakpoints” at any stages of the pipeline that may fail, and only proceeding to the next stage after quality control of the results and possible manual intervention if deemed necessary. Adding in support for manual steps in pipelines could be a useful feature in future versions.

Our other future work will involve improved job management and debugging of the pipelines. Currently when a pipeline has a problem from one of the command line executables, the BatchMake scripts, or the HTCondor grid environment, these can only be debugged using command line tools. The Rodent plugin tracks the output files for each of the command line executables, but does not present them to the user through the web UI. What would be desirable is for the overall DAG of the pipeline to be presented to the user as it is executing. The Rodent pipeline currently uploads results after each command line executable has been run, but does not notify Midas at the start of each executable. If each command line executable was tracked before it starts, as it is processing, and when it completes, the graphic of the DAG could be updated as processing occurs. The standard out and standard error of each command line could be presented to the user, as could the HTCondor log for each executable. Status updates could be presented to the user as color-coded indicators on each of the individual DAG nodes, so the user could see at a glance which nodes have completed, which have yet to run, which are currently running, and the status of completed runs. Since the HTCondor system provides a.dot representation of each DAG, one possibility would be to export this SVG and then use this as a basis for web interaction, using a library like D3^17^. The user could then drill into any individual node to see the logs and outputs, so if a node has an error the user could immediately formulate an understanding of what happened with the command line executable on the HTCondor grid all from the web interface. This process would also allow for more rapid development of pipelines as debugging would be eased and centralized. As result files are added back into Midas, their provenance could also tracked so that the set of operations and parameters leading to their creation will be linked to these outputs, further enhancing the reproducibility of these pipelines. Ideally, this information would be exportable according to the w3c provenance standard^18^, and our future work will incorporate explicitly tracking provenance of all data throughout its lifetime using this standard. Provenance is currently implicitly tracked through the system, but would have to be reconstructed via examination of parameter files constructed for pipeline runs. Systematic quality control is also crucial for a scientific study, and while this current system has no built in mechanism for anomaly detection, this could be added as a step of an image processing pipeline, and is a recommended practice. For example, for diffusion MRI data, DTIPrep could be used for quality control of input data, including both adherence to study protocols and image artifact detection and removal (Farzinfar et al., 2013). Future versions of this work will include an API to propagate quality control measures of the pipelines to the UI and to the pipeline job management system.

The system we have developed in Midas allows for the creation of easy-to-use image processing pipelines, even for those without image processing expertise. These pipelines can be run multiple times by different investigators, but still retain the features of reproducibility because the input data, the parameters and the computing environment is shared and centralized, which prevents reliance on human memory and informal communication. By using Midas, sharing data between collaborators is both safe and simple. Clinical researchers who would not currently have the ability to run complex command line image processing pipelines can now repeatedly run these pipelines on shared datasets, and can share results and intermediates with their collaborators. This simplifies the process of debugging, as when one researcher finishes a pipeline, they can let an image processing expert collaborator know, and even if that expert is in a remote location, viewing the exact results of the pipeline along with the specific inputs and parameters is possible through the centralized Midas server. If the image processing expert makes a fix or change to the executables of the pipelines, they will know that once the pipeline runs correctly for them in Midas, it will run the same for their clinical research partner, and there will not be any problems in terms of dissimilar computational environments or dependencies. Overall this system improves on the rapidity of development time and execution time for complex image processing pipelines and allows for greater reproducibility of experiments, which is one of the fundamental building blocks of scientific research.

### Recommendations for creating automated image processing pipelines

Regardless of whether this particular technology is adopted, our experience has allowed us to explore creation of automated image processing pipelines and to create recommendations to anyone endeavoring to do the same. One of the largest sources of complexity in this work was in trying to allow for automated UI generation by our image processing collaborators, where the UI would be displayed and processed within Midas. We tried to create a complete set of building blocks that could be easily strung together, but as we started each different pipeline, we realized that new and specific building blocks would be needed. Because these building blocks needed to exist within Midas, we could not rely on generic web techniques or components. Providing a clear set of APIs for Midas and for a server side processing framework would allow an interface to be built completely separate from either of these, but that could call into and take advantage of the strengths of both, without becoming overly burdensome on the UI developer or on Midas. To build automated pipelines, the following API component areas are needed, and ideally should be built independently to enable a clean separation of concerns:

- Data management.
- Pipeline and pipeline job status tracking.
- Grid execution, and the interface between applications and the grid environment.

Then a UI can be built using these APIs in whatever web technology is appropriate for the interface developer.

Another large source of complexity was in developing and debugging the pipelines. If each of the individual steps in a pipeline are created supporting the properties listed below (which can also be thought of as the rough sketch of the API for a Pipeline Step, or a Pipeline), they can be developed and tested as independent steps, then combined together into a pipeline. This setup also allows for the pipelines to be run and intermediate results to be examined, parameters changed and steps rerun, allowing the pipelines to be executed in an interactive fashion, with granular control allowed over how much of the pipeline executes asynchronously. These properties are:

- Input files and parameters.
- Output files and output parameters (e.g., scalar values).
- Visualization of input and output files.
- Current status of executable process in the grid.
- Return code of executable.
- Standard out of executable.
- Standard error of executable.
- Log of job execution from grid system.

A pipeline can then be composed of these steps, and the pipeline should have similar information stored, along with a display of the status of the individual pipeline steps and overall status of pipeline execution. Imagine being able to see at a glance the status of the overall pipeline and each step, and to allow the user to drill into the details of a particular step to enable debugging of command line executables through the web UI. Designing a framework with these ideas in mind would allow for flexible and rapid development of automated image processing pipelines.

### Conflict of interest statement

The authors declare that the research was conducted in the absence of any commercial or financial relationships that could be construed as a potential conflict of interest.

## Footnotes

^1^http://www.slicer.org.

^2^http://fsl.fmrib.ox.ac.uk/fsl/fslwiki/.

^3^http://www.fil.ion.ucl.ac.uk/spm/.

^4^http://nipy.sourceforge.net/nipype/.

^5^http://www.midasplatform.org.

^6^http://www.php.net/.

^7^http://www.zend.com/.

^8^http://httpd.apache.org/.

^9^ http://www.mysql.com/.

^10^http://www.postgresql.org/.

^11^http://lucene.apache.org/.

^12^http://www.ics.uci.edu/~fielding/pubs/dissertation/rest_arch_style.htm.

^13^https://github.com/midasplatform/pydas.

^14^http://github.com/midasplatform/rodent.

^15^http://batchmake.org/.

^16^http://research.cs.wisc.edu/htcondor/.

^17^http://d3js.org/.

^18^http://www.w3.org/TR/prov-overview/.
